# Cervical cancer in Nepal: Current screening strategies and challenges

**DOI:** 10.3389/fpubh.2022.980899

**Published:** 2022-11-17

**Authors:** Mohan Narasimhamurthy, Santhosh Upadhyaya Kafle

**Affiliations:** ^1^Department of Pathology and Laboratory Medicine, Pennsylvania Hospital, University of Pennsylvania Health System, Philadelphia, PA, United States; ^2^Department of Pathology, Birat Medical College Teaching Hospital, Tankisinuwari, Nepal

**Keywords:** cervical cancer, Nepal, screening, prevention, HPV

## Abstract

Nepal has a high burden of cervical cancer primarily due to a limited screening program. Most present with advanced cervical disease. Despite no national cervical cancer control program, Nepal's Ministry of Health and Population has taken many initiatives with various international collaborations in screening, vaccination, and treating pre-invasive and invasive cancer. However, the existing prevention and treatment modalities are dismally inadequate to meet the targets of WHO's cervical cancer eliminative initiative by 2030. We provide an overview of the Ministry of Health and Population, Nepal's efforts to tackle the growing cervical cancer burden in the country. We discuss the challenges and potential solutions that could be practical and augment screening uptakes, such as single-dose vaccination and HPV DNA tests. The screen-and-treat approach on the same day could potentially address treatment delays and follow-up loss after testing positive. Our narrative summary highlights existing and innovative strategies, unmet needs, and collaborations required to achieve elimination across implementation contexts.

## Introduction

Nepal is a South Asian landlocked country located between India and Tibet Autonomous Region of China, with an approximate population of 29 million (1). The country is administratively divided into seven provinces which in turn into 77 districts. Nepal's health care providers without robust and effective primary, secondary, and tertiary health referral systems are the public and private health sectors unequally spread across the nation's hilly to low plain regions. The gross domestic product per capita stands at USD 1,222.9 in 2022, 12.7% higher than in 2017 (2). In 2019, 17.4% of Nepalese were multi-dimensionally poor—just under five million persons (3). The estimated life expectancy at birth is 71 years as of 2017 (4), 2 years more than neighboring India. These numbers point toward Nepal's relentless pursuit of progress guided by the overarching national aspiration of “Prosperous Nepal, Happy Nepali” by 2030 (5).

Cervical cancer was the leading cause of death in women in most countries in the middle of the 20th century. Since the introduction of the Papanicolaou (Pap) test, cervical cancer has dramatically reduced in high-income countries over the last five decades (6). In contrast, cervical cancer remains and is rising among women in low- and middle-income countries (LMICs), with an estimated 531,631 (88%) of 604,127 new cases yearly. Within Nepal, Cervical cancer continues to be the leading cancer among women, with an annual incidence of 2,244 new cases and 1,493 deaths. Nepal has a cervical cancer incidence of 16.4 per 100,000 women, in contrast to the WHO's desired target of 4 per 100,000 women, nearly four times the target to eliminate the public health issue of cervical cancer (7). In Nepal, cervical cancer kills almost 11 women for every 100,000, even though cervical cancer is preventable with time-tested screening strategies (1). In addition to the toll on health and mortality, cervical cancer imposes a significant social and economic burden in LMICs like Nepal. The WHO estimates that by investing in cervical cancer prevention and control, nations can empower an estimated 250,000 women to contribute to the world's economy, which is estimated to be $28 billion through 2050 (8).

The WHO recognizes the enormous cervical cancer burden on women living in impoverished low middle-income countries. Hence, to address this global health burden, the WHO launched “The Global Strategy for the Elimination of Cervical Cancer,” with an intermediate 2030 triple-intervention strategy known as the 90–70–90 targets in 2019. The triple targets to achieve are 1-vaccination of 90% of girls with the HPV vaccine by age 15, 2-screening of 70% of women by 35 and 45 years of age, 3-Treatment and management of 90% of women identified with the cervical pre-invasive and invasive cancer respectively. The successful implementation and scaling-up of the triple intervention would reduce mortality attributed to cervical cancer by 33.9% (24.4–37.9 per 100,000 women) by 2030 and almost 99% by 2120 (mortality) among women aged between 30 and 69 years (8).

Globally, as nations embrace the WHO's call to action on the cervical cancer elimination initiative, we sought to summarize the current status of various screening strategies and treatments available for cervical cancer in Nepal and the challenges to surpass the WHO's 90–70–90 targets.

## Screening strategies

Cervical cancer development is a multistep carcinogenic process and starts with HPV infection. Dr. Hausen, in 1975 hypothesized the link between cervical cancer and Human Papillomavirus (HPV) (9). The following decades witnessed rapid progress in understanding the pathogenesis of HPV-driven cervical cancer. More than 200 HPV genotypes have been identified, subdivided into low-risk and high-risk categories based on their pathogenicity to cause cancer. Once HPV infection occurs, it can regress, persist or progress (10). Persistent high-risk HPV infections, most commonly 16 and 18 genotypes, are responsible for nearly all invasive cervical cancer (11). High-risk HPV 16 and 18 elaborate oncoproteins E6 and E7 implicated in carcinogenesis: E6 binds to p53, accelerating its degradation, while E7 binds to pRB, releasing E2F, which allows cells to progress in the cell cycle with genomic instability (12–14). In addition, the integration of viral genes in the host genome facilitates the further expression of E6 and E7, with subsequent lethal genetic changes contributing to neoplastic transformation. It takes 10–20 years, or even longer, for HPV-infected cells to progress from normal to pre-invasive to invasive cancer. This long interval provides a window to detect early pre-invasive neoplastic lesions and prevent cancer development by screening. There are three methods of cervical cancer screening. They are:

i) Cytology-based screening: conventional pap smear and liquid-based cytology.
ii) Visual inspection by acetic acid examination.
iii) HPV DNA testing concurrent with pap smear (co-testing) or primary screening technique.

### Cytology-based screening

The Pap smear test was developed in the early 1940s by George Papanicolaou. The test involves taking the sample from the cervix and smeared on the glass slide with subsequent staining for microscope examination by a trained cytotechnologist or pathologist. It is the earliest screening technique that became widespread by the 1960s, chiefly in high-income countries with 70–80% sensitivity (15–17). The specimen adequacy is crucial for the Pap test's accuracy (18). The cellular changes are reported according to “The Bethesda System for Reporting Cervical Cytology,” which provides consistent and reproducible criteria for diagnosing pre-invasive and invasive cancer (19). The most important advantage of a conventional pap smear is its low cost (20). A significant advance in cytology-based screening happened when US FDA approved a new liquid-based cytology technique (LBC: Thin prep and SurePath) to enhance further the sensitivity to detect various pre-invasive lesions and improve specimen adequacy (21).

In contrast to a conventional pap smear, the collected sample is placed inside a preservative liquid in a small bottle of LBC. At the laboratory, mucus and blood were removed, and cells were placed on a glass slide for microscopic examination. The distinct advantages of LBC are fewer unsatisfactory smears, high sensitivity, less obscuring materials such as blood, mucous, and inflammatory cells in smears, and residual cell suspension for testing human papillomavirus (HPV) DNA (22). Though liquid-based cytology is available in the selected private laboratories, it is not a common technique used in the public sector in Nepal. A recent systematic review and meta-analysis by Shrestha et al., which included 17 studies from Nepal, reported that liquid-based cytology is not a screening method in Nepal (7).

High-income countries witnessed the impact of cytology-based screening, which is the most successful screening technique for cancer prevention ever designed. Nearly 90% of women were screened at least once in their lifetime in these countries, attributed to organized quality-assured screening programs and widespread public awareness (23). Because of the successful national cervical cancer screening programs, high-income countries are on the verge of cervical cancer elimination (24). Without such a screening program, only 2.8% of Nepali women were screened when the population at risk is 11.4 million women aged 15 years and older (25). The most vulnerable are women (>80%) living in rural areas. Screened women were mainly from urban areas highlighting the further inequality in accessing the available health services (25). There is no data available on whether these screened women were asymptomatic or symptomatic such as irregular and postcoital bleeding and vaginal discharge, which could guide us in the screening uptake behavior among urban women.

The cytology-based screening process is a highly skilled personnel-intensive program. Major limitations are the low sensitivity to detect early pre-invasive lesions, the complex logistical and care network to implement quality control and subsequent appropriate clinical management (e.g., colposcopy, biopsy, endocervical curettage) of women with positive screening. These reasons precluded low- and middle-income countries, including Nepal, from rolling out population-based screening, where screening occurs opportunistically in health camps (26–28). Therefore, implementing cytology-based screening to enhance the coverage from the current 2.8% to the WHO target of 70% by 2030 seems remote in Nepal. However, the Ministry of Health and Population, Nepal, must continue investing in cytology which is of immense value for triaging women who test hrHPV positive by any other techniques.

### See and treat approach

An alternative cost-effective screening technique is a visual inspection of the cervix by applying 3–5% acetic acid (VIA). The application of acetic acid highlights the cervical dysplastic areas with immediate color changes visible to the naked eye. Any modification in the color is categorized as positive for pre-invasive cervical cancer. VIA is a simple, easy-to-use technique that has been used since the 1990s, especially in LMICs, including Nepal. The distinct advantages of the VIA technique are that healthcare personnel can perform without requiring high technology or infrastructure and the same-day “Screen and Treat” (SAT) strategy. Previous studies have shown that a single-visit approach effectively reduces high cervical precancerous lesions (29). Low and middle-income countries have implemented SAT strategy in pilot programs and sporadically with much success (30–32). Several studies have found the sensitivity of VIA for detecting high-grade cervical pre-invasive lesions ranges from 73 to 85% and a specificity of 81–89% (33–35). The VIA technique's drawbacks are provider-dependent and subjectivity and have lower sensitivity for women older than 40. However, the benefits outweigh these drawbacks in the current situation.

The cervical cancer burden in Nepal has not gone unnoticed by the Government of Nepal. In 2010 The Ministry of Health and Population, Nepal developed “national guidelines for cervical cancer screening.” The guidelines envisioned screening 50% of the target population, women in the age range of 30–60 years, by 2015 based on the VIA technique. However, the screening program did not gain momentum resulting in dismally low coverage owing to implementation difficulties. Recently, a pilot study was initiated to investigate the Effect of a “community-based intervention for cervical cancer screening uptake in a semi-urban area of Pokhara Metropolitan, Nepal” (COBIN-C) (36). This study is based on trained female community health volunteers (FCHVs), important last-mile connectivity to the community (37) who deliver home-based health education to enhance the cervical cancer uptake by VIA technique among eligible women. The study results are crucial and expected to shed light on the social and cultural barriers, community health practices, and how the intervention results in overcoming the obstacles to a positive attitude toward cervical cancer screening.

The Ministry of Health and Population, Nepal, issued “The national guideline for Cervical Cancer Screening and Prevention (CCSP)” in 2010. The goal was to screen at least half of women in the age group of 30–60 years, which was revised to 70% in 2017. By 2019, only 8.2% of women aged 30–49 years were screened.

### HPV/DNA-based molecular test as a primary screening assay

Human papillomavirus (HPV) is a small, non-enveloped deoxyribonucleic acid (DNA) virus belonging to the Papillomavirus family and the most common sexually transmitted infection. HPV is highly transmissible, with peak incidence soon after the onset of sexual activity, and most persons acquire infection at some time in their lives. A deep understanding of HPV biology led to the development of HPV-based diagnostic tests and HPV vaccines (38). Among 200 HPV genotypes, 40 are known to infect the genital tract determining cervical carcinogenicity. The genotypes with greatest risk are HPV 16, 18 followed by 31, 33, 35, 39, 45, 51, 52, 56, 58 and 59. HPV types 16 and 18 account for 73% of cervical cancers globally (39). The studies have shown regional differences in the prevalence of HPV genotypes associated with cervical cancer (40). The reliable data on the prevalence of HPV genotype in Nepal would greatly help to consider the scope of HPV DNA testing and HPV vaccination tailored to its locoregional needs. To this end, two studies in Nepal demonstrated that overall HPV prevalence was 8.6%, and 90% of examined cervical samples showed high-risk genotypes HPV 16 and 18. HPV 16 and 18 infection is 2% among Nepalese women and is responsible for 80.3% of invasive cervical cancers (41, 42).

In 2002, the American Cancer Society (ASC) guidelines incorporated HPV DNA testing in screening pre-invasive cervical cancer in tandem with cytology (43). Further research with accumulating data showed that primary cervical cancer screening by HPV DNA testing was comparable to cytology alone or co-testing in addition to longer screening intervals (44–47). In 2014, HPV DNA testing became a primary screening test in addition to cytology when the US FDA approved the Cobas HPV test as a first-line screening test for 25 years and older women (48). Recent studies across the globe, including LMICs, have shown that HPV testing is a reliable, reproducible, and cost-effective screening compared to cytology screening for cervical neoplasia (49–53).

WHO recommends screening should start at the age of 30 years with regular testing every 5–10 years for women. In contrast, women with HIV should begin screening at 25 years, with regular screening every 3–5 years.

#### HPV DNA-based screening tests

Initial HPV DNA tests were polymerase chain reaction (PCR) based nucleic acid amplification tests (NAAT) or signal amplification techniques, such as the Digene Hybrid Capture^®^ II assay (38). A recent study from Srilanka successfully demonstrated that HPV-DNA testing using Cobas 4800 HPV/DNA automated PCR machine can be implemented as a primary screening method in low-resource settings (49). In recent years, the development of rapid molecular-based point-of-care tests for detecting HPV DNA (e.g., care HPV^®^–Qiagen, GeneXpert^®^–Cepheid) has outperformed the earlier expensive, time-consuming, and laboratory-intensive techniques (54). In a study in a rural Chinese population, the authors found primary HPV DNA Qiagen testing compares favorably to VIA cost-effectively (55). These accurate and affordable rapid tests provide new options to roll out mass cervical cancer screening programs in Nepal (52).

#### Advantages and limitations of HPV DNA-based screening tests

The distinct advantages are cost effectiveness, suitability for all settings, reduced investment in health workforces and infrastructure, and prolonging the screening interval. DNA-based tests also leave no space for human errors, such as subjective and interobserver variability associated with pap smear and visual inspection methods (56). For these reasons, WHO recommends hrHPV testing as a preferred screening strategy wherever feasible (57, 58). The flip side of this argument is that HPV-DNA test as a primary screening could potentially detect clinically insignificant diseases than the women at risk of developing cervical cancer because it is highly sensitive but less specific than cytology alone (38). Therefore, triaging to determine the optimal management of HPV-DNA test-positive women is essential to avoid unnecessary diagnostic and treatment burdens on the health system, outweighing the benefits of HPV DNA testing.

### Barriers to cervical cancer screening programs in Nepal

Barriers are not significantly different across low and middle-income countries. However, there are many locoregional specifics, such as hilly regions of Nepal rendering the accessibility to the health facility. In general, we can break down barriers into three major categories.

#### Clinic and laboratory

Setup is a bare minimum for any of the three screening methods to succeed. According to a recent report by Nepal's Ministry of Health and Population, the laboratory testing capacity in Nepal is that only 12% of facilities can perform basic tests like hemoglobin, malaria testing, and stool microscopy (4). Despite sparse physical infrastructure, there are stories of sporadic success when planned well.

#### Cultural and social factors

Besides the economic sustainability, numerous studies have highlighted the challenges for nationwide cervical cancer screening in Nepal. Institutional research at a tertiary care center in Kathmandu showed varying knowledge of cervical cancer among participants; 37% had an average, and 16.5% had good knowledge. Further, 70% of the participants had a positive outlook toward cervical cancer screening. Surprisingly, the cervical cancer screening uptake among those with a positive outlook was < 25%. The significant barriers to screening were, in descending order, embarrassment (72%), pain (71%), lack of privacy (65.9%) (59), and misconceptions about the screening. Additional obstacles were social issues, cultural barriers, healthcare workers' behavior, and geographical challenges in seeking screening center services.

#### Financial sustainability

Economic assessment of any screening strategy has enormous implications for the success in the context of LMICs, including Nepal. Each country should develop its economic evaluation. In addition, on behalf of LMICs, WHO must negotiate global pricing of HPV testing with the manufacturers. The prices should be competitive for the LMICs so the governments can pay. Innovative and sustainable financial models for procuring HPV tests should be developed to enable those most in need to access the tests.

### Steps to be taken and emerging techniques to overcome the known barriers

For LMICs like Nepal, the most feasible screening technique is the hrHPV molecular assay. hrHPV testing can be done on a self-collected sample. Self-sampling for HPV is an innovative technique to collect the specimen in privacy with many advantages: women can decide their time, place, and comfort level. It can potentially overcome the fear and stigma associated with visiting the clinic, a trained clinician, and a pelvic examination (60–62). In addition, the self-sampling specimens are comparable with the provider-taken specimen, and samples can be stored for up to 32 weeks for later transportation without compromising specimen quality (63). Thus, the self-sampling technique offers critical advantages in successfully overcoming the known barriers and implementing Nepal's cervical cancer screening program.

A meta-analysis by Arbyn (64) found that participants' acceptance of self-sampling for HPV screening was two times more than women without. The acceptance rate went further high when women received the HPV self-sampling kits at home either by mail or from a health worker. Shrestha et al. (65) explored the concept of self-sampling for HPV DNA testing among Nepali women in Kathmandu Valley, mostly limited to urban areas, demonstrating that 56.7% of the participants were willing to accept the self-sampling technique. Because of the disparity between urban and rural Nepal, the results cannot be generalized. In a recent study in rural, southwestern Uganda, authors studied the challenges associated with “implementing community-based human papillomavirus self-sampling with SMS text follow-up for cervical cancer screening.” 82% of eligible women underwent self-sampling hrHPV testing. Most women rated self-sampling highly and confidence in test results was higher for self-screening than VIA. Despite good acceptability, only 35% hrHPV positive women returned for follow-up despite SMS texts. This study identified the gap in the cervical cancer screening cascade and linkage to care (66).

Successful implementation of HPV self-sampling screening programs in Nepal depends on how we address the above challenges, such as identifying the target population, educating them about the technique, and removing the fear from their mind and immediate family. WHO has created a great resource highlighting the factors to consider while introducing HPV self-sampling. The success of self-sampling-driven HPV DNA testing in Nepal largely depends on the active participation of the Female Community Health Volunteers (FCHV). They have a strong presence in all 77 districts of the country. According to the family health division under the Ministry of Health and Population, Government of Nepal, there are 47,328 FCHVs at the rural level and 4,142 at the urban level establishing last-mile community connectivity (4). Currently, FCHVs are actively involved in advocacy, promotion, and service delivery with an overall aim to support maternal and child health, family planning, and other community-based health activities. Expanding and involving FCHVs in cervical cancer elimination initiatives is imminent if we need to succeed in our efforts to reach out to all eligible women for screening by 2030. The Nepal government should support the FCHV by empowering them with knowledge, skills, and training to increase awareness of cervical cancer screening among community members. The other significant challenges to address while implementing the self-sampling technique are; options for returning the sample and; receiving the test results. The next big challenge is triaging women with positive results. The system should not allow positive cases to slip out of the radar for clinical assessment and treatment of cervical lesions. We are optimistic that the study results of the COBIN-C trial will address this challenge.

In the future, screening by cost-effective HPV testing alternative to cytology and VIA will auger well in LMICs. Policymakers should consider HPV testing with self-collection samples as it gains traction among the population (51).

Cytology has been the mainstay for cervical cancer screening for decades and has been largely successful in reducing cervical cancer in high-income countries. However, screening strategies are changing, with many different options available now. Current risk-based management is largely based on established practice from cytology-based screening programs. Hence, evaluating the different cervical cancer prevention options in risk-based management is critical moving forward.

## HPV vaccination status, success, and challenges

The tremendous progress in understanding HPV and cervical cancer's natural history has allowed primary prevention by vaccination against HPV to become a reality. The vaccine is primarily used to avoid an HPV infection; thus, its administration before the onset of sexual activity gives the best chance of preventing the disease. The first milestone in that direction was in 2006 when GlaxoSmithKline produced an AS04-adjuvanted HPV-16/18 bivalent vaccine (Cervarix) that has proven effective in preventing HPV-16/18-related persistent infections and cervical intraepithelial neoplasia grade 2 and above (67).

Subsequently, Merck produced Gardasil, a quadrivalent vaccine against HPV types 6, 11, 16, and 18 (68); and Gardasil-9, a non-avalent vaccine for HPV types 31, 33, 45, 52, and 58, in addition to the coverage of quadrivalent vaccine (69). All three HPV vaccines when administered intramuscularly, have resulted in good immunogenicity with persistent high anti-HPV antibody titers in adolescents (aged 9–15 years, two doses) and young women (16–26 years, three doses). All the vaccines were well tolerated, without any major vaccine-related adverse events.

The HPV infection among sexually active women is almost two-thirds, and hrHPV 16 and 18 genotypes are responsible for more than two-thirds of cervical cancer cases (70, 71). HPV vaccines have been approved for women in developed countries since 2006. In contrast, access to new vaccines in developing nations has historically been a decade late; however, HPV vaccines are now available in at least 124 countries, including Nepal. Only 15% of girls in the target age for HPV vaccination are globally fully protected (72). For complete protection against cervical cancer, WHO recommends two doses of HPV vaccine for 9 and 14 years, aged girls. However, only about 15% of eligible girls worldwide have been fully vaccinated (71). Several studies highlighted common factors across low–middle-income countries that led to such low coverage; high cost, supply chain hurdles, and lack of national HPV vaccination programs.

### Current vaccination status in Nepal

The median age range of first sexual activity is 16.5–17.9 for women in Nepal (73, 74).

The first attempt at HPV vaccination in Nepal was carried out in 2008 using 3,300 vials of Gardasil with the assistance of the Australian Cervical Cancer Foundation (ACCF) (75). Seventeen schools were selected; 1,096 school girls aged 10–26 were vaccinated; 90% were 12–16. Only five and two girls missed their second and third doses, respectively, making it a highly successful vaccination drive. The success of this collaboration also led to the establishment of the Nepal Australian Cervical Cancer Foundation (NACCF), which has been a strong advocate of public awareness at the community level and provides free-of-cost vaccines. In addition, collaboration with GAVI led to the HPV vaccine demonstration project in 2016–2017, launched in Chitwan (8,243 girls) and Kaski (6,500 girls) districts. This project incorporated the two doses of Cervarix vaccine into the annual regular immunization program for girls between 11 and 13 years at school and 10 years old out-of-school girls at the health facility. Encouraged by the success of the pilot projects, the Nepal Government launched an HPV vaccination drive in nine districts across the country. Regrettably, the prevailing political scenario and lack of funds derailed the vaccination drive and halted it indefinitely (76). Currently, Nepal does not have a national HPV vaccination program; hence no vaccination coverage data in the country.

### Barriers to HPV vaccination and strategies to roll out a national HPV vaccination program

Previous vaccination programs in Nepal have demonstrated that HPV vaccination acceptance was high among school-going girls despite less knowledge of HPV; only 13.9% knew of an HPV vaccine. In one study, 96% of parents expressed willingness to have their child HPV vaccinated if it is free of cost. The high vaccine cost seems to be the most significant barrier to achieving WHO's target of 90% by 2030. Financial sustainability is crucial for introducing and scaling up an HPV vaccination program. Recently approved “the quadrivalent Cervavac vaccine” in India costs ~400–500 Nepali rupees (USD 5) per dose compared to the currently available vaccines for USD 46 per vaccinated person (76). Another bivalent vaccine, Cecolin, which has been licensed in China, is presently under an active prequalification process by WHO making them promising more affordable vaccines than existing licensed HPV vaccines (77). Another encouraging piece of information is the New England Journal of Medicine data, which could further reduce the cost and vaccine affordability (78). Additionally, the WHO Strategic Advisory Group of Experts on Immunization (SAGE) concluded that a single-dose Human Papillomavirus (HPV) vaccine is comparable to 2-dose schedules in its efficacy (79). Single-dose HPV vaccine administration simplifies the system, which is logistically less expensive, easy to administer with broader coverage rates, and ideally suited for LMICs like Nepal.

SAGE recommends updating dose schedules for HPV as follows:

- One or two-dose schedule for the primary target of girls aged 9–14.
- One or two-dose schedule for young women aged 15–20.
- Two doses with a 6-month interval for women older than 21.

There is limited evidence regarding the efficacy of a single dose in immunocompromised individuals, including those with HIV, who should receive three doses if feasible, and, if not, at least two doses.

In addition, social and cultural factors like ethnic variations, public awareness, reaching out to non-school-going girls, consent issues, and strong political commitment are the major hurdles to launching and implementing the vaccination program in Nepal (59, 80). Vaccine hesitancy in the pilot programs is a non-factor in the vaccination uptake among school-going girls in Nepal. These data will provide relevant evidence to plan Nepal's following cervical cancer prevention programs. Cervical cancer will remain problematic unless an effective HPV vaccine program is rolled out to all adolescent girls, irrespective of social and economic status. Concerted and well-coordinated efforts between the ministry of health, Nepal, its partners, and the private sector are essential to overcome seemingly possible hurdles. One shining example of these efforts is a Sub-Saharan African nation: Rwanda has rolled out the comprehensive cervical cancer program and had incredible success in the HPV vaccination coverage across the nation. The coverage rate is comparable to the high-income countries and is on the verge of eradication (81). A robust vaccination strategy and human resource framework led to this spectacular success in Rwanda.

The recent Covid-19 pandemic has shown that the governments in low-middle-income countries have the ability and political will to administer an enormous number of vaccines to their public. Ministry of Health and Population, Nepal must reach out to the neighboring countries to procure the Cervavac and Cecolin vaccines at a negotiated price as it did to get the Covid-19 vaccine. Nepal government must change the protocol to a school-based immunization program incorporating the HPV vaccine ensuring high coverage among young girls aged 9–14. Additionally, the inclusion of HPV vaccination and cervical cancer screening within community-based immunization programs that provide sexual and reproductive health, human immunodeficiency virus (HIV)/sexually transmitted infection (STI) screening, and management is feasible.

## Cervical cancer treatment

In Nepal, 2,244 new cases of cervical cancer are diagnosed annually, and 1,493 women die of cervical cancer (74). Notwithstanding the prevention interventions, these new cervical cancer cases will impact the next 10–20 years. Hence, priority must be on early detection of precancerous and treatment of invasive cancer. Mostly, women with cervical cancer have locally advanced disease at diagnosis, requiring radiation and cisplatin-based chemotherapy rather than surgery (82). Nepal's healthcare personnel trained to manage such cases is severely limited. Specialized physicians are few: estimated to be 20 gynecologic oncologists and 35 radiation oncologists in the country, catering to 29 million populations. They are mainly concentrated in urban centers. The total strength of healthcare providers trained to manage cervical pre-invasive lesions is unknown.

A historical city Banepa saw the first women's clinic, constructed by NACCF with the support of ACCF. It is a model health center in Nepal, providing care for VIA+ women and offering general screening and examination facilities. Skilled human personnel specialized in performing VIA, colposcopy and cervical biopsy, thermal ablation, and loop excision are acutely short in Nepal. Though infrequent, a recent visit by a team of experts from the USA comprising the members of The American Society of Clinical Oncology (ASCO) and The University of Texas MD Anderson Cancer Center trained 42 personnel in essential skills for the diagnostic procedure and management of cervical cancer (83). Appropriate cancer care requires a multidisciplinary approach with a team of experts consisting of oncologists, surgeons, pathologists, radiologists, oncology/radiation nurses, medical physicists, radiation therapy technicians, and trained social health workers. Local governments have partnered with various domestic and international stakeholders such as the Bill and Melinda Gates Foundation, PATH (a global health organization), Global Alliance for Vaccine and Immunization, and ASCO to recruit, train and retain health care personnel to mitigate the effects of health care personnel shortage.

## Integration of the lessons learned into the existing health infrastructure

A lack of medical knowledge and reluctance to seek timely healthcare contribute to the cervical cancer burden in Nepal. However, the enthusiasm from participants for sporadic attempts at screening and vaccination in Nepal is encouraging. With the growing Mobile health (mHealth) technology, the Ministry of Health and Population, Nepal, can reach out to every corner of the country with health campaigns. Recent experience with MANTRA, a mobile game app in rural Nepal, developed to tackle maternal and child health issues, demonstrated positive engagement with rural women despite limited educational level (84). It enabled them to identify the early danger signs and make informed health decisions. FCHVs were encouraged by the participant's responses, and they acknowledged that MANTRA intervention amplified the impact of their efforts in rural Nepal.

Nepal has higher mobile subscriptions than most countries in South Asia, with ~110 subscriptions per 100 people, according to World Bank's 2016 data (85). This should enable mHealth interventions to be easily incorporated into Nepal's existing national health infrastructure, which begins with health posts. Health posts are Nepal's first institutional contact point for basic health services. These bottom-level health facilities monitor the activities of female community health volunteers (FCHVs). Primary health care outreach clinics (PHC-ORCs) and Expanded Program on Immunization (EPI) are additional basic health services. Each level above the health post level is a referral point in a network ranging from primary health care centers (PHCCs) to primary- and secondary level hospitals and, finally, tertiary-level hospitals. Community health units are gradually increasing at the ward level. In addition, Nepal has established urban health centers (UHCs) to ensure that the urban poor can receive treatment.

In summary, Nepal's efforts to eliminate cervical cancer must be sustainable and continuous. Widespread single-dose HPV vaccination and point-of-care (POC) HPV testing with self-sampling should form the basis for Nepal's national cervical cancer screening program. Pro-active involvement of FCHVs, in the above strategy, must be initiated. Follow-up care for women who tested positive should be provided in the designated clinics backed by a robust reflex communication system for recall reminders. Further, the knowledge and evidence from the previous and ongoing efforts should guide the Nepal government's policymakers about the necessary domestic and international collaborations, which will augment the capacity secondary prevention and management of cervical cancer with a reliable infrastructure to treat HPV-driven pre-invasive and invasive cancer.

## Author contributions

MN: conceived, literature search and review, and manuscript writing. SK: manuscript review and extensive input on-ground facts. All authors contributed to the article and approved the submitted version.

## Conflict of interest

The authors declare that the research was conducted in the absence of any commercial or financial relationships that could be construed as a potential conflict of interest.

## Publisher's note

All claims expressed in this article are solely those of the authors and do not necessarily represent those of their affiliated organizations, or those of the publisher, the editors and the reviewers. Any product that may be evaluated in this article, or claim that may be made by its manufacturer, is not guaranteed or endorsed by the publisher.
